# Anthropometric differences among natives of Abuja living in urban and rural communities: correlations with other cardiovascular risk factors

**DOI:** 10.1186/1756-0500-6-123

**Published:** 2013-03-27

**Authors:** Olufemi Sola Adediran, Philip Babatunde Adebayo, Adeseye Abiodun Akintunde

**Affiliations:** 1 Department of Medicine, College of Health Sciences, Benue State University, Makurdi, Benue State, Nigeria; 2 Departments of Medicine, Ladoke Akintola University of Technology/Ladoke Akintola University of Technology Teaching Hospital, Ogbomoso, Oyo-State, Nigeria

**Keywords:** Anthropometry, Cardiovascular risk factors, Nigeria, Rural, Urban

## Abstract

**Background:**

There is an increase of obesity and other cardiovascular risk factors worldwide, but especially in developing countries where multifaceted transitions are occurring. There is need for more evidence for the cardio-metabolic effect of changing lifestyles and urbanization in Nigeria. This study aimed at defining rural–urban differences in anthropometric parameters in two Nigerian communities of the same ancestral origin and to determine the cardiovascular risk correlates of these anthropometric measurements. This was a cross-sectional epidemiological study using stratified cluster sampling method. We studied 335 and 332 urban and rural dwellers respectively. A complete cardiovascular profile as well as anthropometric measurements was compared between the two populations.

**Results:**

All anthropometric indices considered in this study (weight, BMI, waist circumference, waist circumference/height ratio, abdominal height; biceps, triceps, sub-scapular, abdominal, superior iliac skinfold thicknesses) were significantly higher in urban than in the rural population (p = <0.001). Overweight, obesity and hypertension were significantly prevalent among the urban population (p = <0.001) while there was no significant difference in the prevalence of dyslipidaemia (p = 0.096) and diabetes (p = 0.083) between the two cohorts. Females tend to have a higher chance of obesity than males although there was no gender difference in waist circumference and central skin fold thickness in the rural population. Age was the significant predictor of systolic blood pressure among the rural (R^2^ = 0.157, β = 0.258, p = 0.016) and urban female population (R^2^ = 0.201, β = 0.351, p = <0.001) while Abdominal height (R^2^ = 0.16, β = 0.281, p = 0.001) and waist circumference (R^2^ = 0.064 β = 0.064, p = .003) were predictors of systolic blood pressure in urban and rural men respectively.

**Conclusion:**

Anthropometric indices were significantly higher among the urban than the rural populations. Cardiovascular risks were equally more prevalent among the urban population. Appropriate health education and lifestyle modification strategies may reduce the increased burden of cardiovascular risk factors associated with rural–urban migration.

## Background

The growing prevalence of overweight and obesity worldwide has driven an increase in the cases of diabetes and hypertension-which are the major cardiovascular risk factors especially in the developing world [1]. The consequences of this surge are especially felt in developing countries where the old scourge of malnutrition; underweight and infectious diseases already present a ubiquitous health challenge [1]. An explanation for this apparent trend has been hypothesized to be the nutritional, demographic, epidemiological, and socioeconomic transitions occurring in many developing countries [2]. Paradoxically, the shift in the pattern of non-communicable diseases (NCDs) is occurring at a faster rate than it did in the industrialized regions of the world half a century ago [3]. While it is generally accepted that economic development is by and large positively associated with human health [2], ample evidence suggest that this is not always the case, either over the short-term, in relation to booms and recessions [4-6], or over the long-term [7,8].

Rural–urban differences in obesity, the metabolic syndrome, and type 2 diabetes mellitus (T2DM) is seen in most developing countries [9]. Whereas overweight and obesity in underprivileged people in developed countries is substantial, in developing countries rural-based people are mostly lean and have low prevalence of T2DM and cardiovascular diseases (CVD). However, underprivileged people residing in urban areas (mostly rural to urban migrants) show increasing prevalence of overweight/obesity and other cardiovascular risk factors [10]. Although epidemiological surveys in Nigeria have documented the rise in incidence of overweight, obesity as well as the metabolic syndrome as we adopt western dietary and lifestyle pattern [11,12], fewer comparative studies exist to provide evidence for the increasing effect of urbanization [13-15].

Created from three states in 1976, the capital city of the federal republic of Nigeria now has a growth rate four times higher than the average growth rate of other capital cities in Africa [16]. This high growth rate is attributed to many factors among which are; the huge attraction of Abuja due to its combination of excellent infrastructures and facilities [16]. The natives of the present geographical location of the federal capital territory (FCT) Abuja are known as Gbagis, and their main occupation was farming [17]. Following the creation of the FCT, there were several attempts by the government to internalize the natives into the urban life that the new capital offered. This gave birth to several satellite towns around the capital with consequent exodus of some Gbagis to these settlements. An example is Kubwa, a popular satellite town which was quite an enviable place and well equipped with basic amenities. When many of the natives decided to sell their houses to other Nigerians who moved into Abuja, a considerable number relocated back into undeveloped areas of the city, while some joined their kinsmen in Garki village which has the highest population of natives [17].

In the light of the foregoing, we hypothesize that the aboriginal population of the federal capital territory (FCT), Abuja, Nigeria presently living in urban area have developed more cardiovascular risks and higher anthropometric indices compared to their counterpart still living in rural settlements. This study aimed therefore at defining rural–urban differences in anthropometric parameters in two Nigerian communities of the same ancestral origin and to determine the cardiovascular risk correlates of these anthropometric measurements.

## Methods

The study was a cross-sectional, community-based epidemiological study using stratified cluster sampling design conducted in rural Kuseki village in Kuje area council and urban Garki in Abuja Municipal Area Council both in the Federal Capital Territory (FCT), Abuja, Nigeria from August- December, 2010. These populations were the original natives of the area that now bears the FCT. The study area was divided into approximately four equal sectors using the electoral list. The minimum sample size of 174 (in each location) was derived from the formulae n = Z^2^pq/d^2^ where n = minimum sample size, z = confidence limits = 1.96 (95% confidence level), p = prevalence of metabolic syndrome in Abuja (13%) [18], q = 1-p and d = degree of accuracy (0.05). We however chose a sample above the minimum to increase the power of the study and to allow for non-response.

The first house was randomly decided and thereafter every tenth house was taken for the study. If the chosen residents were unwilling to participate in the study, or not a native Abuja resident, the adjacent house was selected. All members of the household with age less than 18 years as well as pregnant women were excluded from the study. The eligible residents in the selected houses were recruited. If desired number of subjects could not be included and the end of the area was reached, investigators returned back to the starting point and the above procedure was repeated until all the remaining subjects were enrolled. The same procedure was applied in all the sectors and sites. All subjects had their sociodemographic variables recorded. They were also assessed for smoking and alcohol consumption. All the subjects were fully informed about the purpose of the study and a written informed consent was obtained from each of them. Approval for the study was obtained from the health research and ethics committee of both local governments as well as the University of Markudi, Benue state, Nigeria.

### Anthropometric measurements

Body weight (to nearest 0.1 kg) and height (to nearest 0.1 cm) were measured while subjects were dressed in light clothing and stood erect with bare foot and eyes directed straight ahead. Body mass index (BMI) was calculated as weight (kg)/ height (m)^2^. Waist circumference (WC) was measured midway between iliac crest. The skinfolds (biceps, triceps, subscapular, suprailiac and abdominal) thickness were measured using Lange skinfold calipers by the same physician (OSA). The sum of all skinfolds (S5SF), central (sum of subscapular skinfold, suprailiac skinfold and abdominal skin fold) and peripheral skinfolds (sum of biceps skinfold and triceps skinfold) were calculated.

### Measurement of percentage body Fat and blood pressure

The percentage body fat (%BF) was estimated using Deurenberg formulae [19] Blood pressure and resting heart rate measurements. Participants were seated for at least 5 min before the diastolic blood pressure (DBP), systolic blood pressures (SBP) and resting heart rate (RHR) were measured on the left arm with arcusson mercury sphygmomanometer. The average of three consecutive measurements was taken as the mean systolic and diastolic blood pressure. To define high levels of blood pressure the recent criteria recommended by the WHO were used; hypertension: SBP of 140 mm Hg and/or DBP of 90 mmHg, or use of blood pressure lowering drugs.

### Biochemical measurements

A fasting venous blood sample was obtained after anthropometry and physical examination for blood glucose and lipid profile. Estimation of total cholesterol (TC), serum triglycerides (TG), and high-density lipoprotein cholesterol (HDL-C), was performed on the sample drawn after 12 hour overnight fast. TC was estimated with the ferric chloride method [20]. The method described by Rosenberg and Gottfried [21] was used for the determination of TG. After precipitation of very low-density lipoprotein cholesterol and low-density lipoprotein cholesterol (LDL-C) from the serum by phosphotungstic acid and magnesium chloride, the supernatant was taken and HDL-C estimation performed by the method described for TC. The value of LDL-C was calculated using Friedwald’s equation [22].

### Definitions

BMI, WC and %BF categories were determined according to WHO criteria, with overweight defined as BMI = 25–29.9 kg/m^2^; WC = 80–87.9 cm in women and WC = 94-101.9 cm in men; and obesity as BMI >30 kg/m^2^; WC > 88 cm in women and WC >102 cm in men [23,24]; For BF%, over weight was defined as BF = 33-39% in women and BF = 21-27% in men, and obesity as BF > 39% in women and BF > 27% in men [23]. Further, S5SF ≥50 mm was taken as high. Impaired fasting glucose (FBS ≥ 6.1 mmol/L) and diabetes (FBS ≥ 7.0 mmol/L) were diagnosed according to the WHO criteria [25]. The National Cholesterol Education Program Adult Treatment Panel III criteria was used to define dyslipidaemia (Total cholesterol ≥ 5.1 mmol/L, triglycerides ≥ 1.7 mmol/L, LDL-C ≥ 3.3 mmol/L and HDL-C ≤ 1.0 mmol/L) [26].

### Statistical methods

Data were recorded on a pre-designed proforma and latter entered into a SPSS spreadsheet. For the variables following approximate normal distribution, mean and standard deviation (SD) were computed, while categorical variables were expressed as frequencies and percentages. Student’s t-test was used to compare the mean values in the two independent groups while categorical variables were compared using pearson chi square test. Correlations between variables were analyzed using the non-parametric spearman rank order test. A multivariate regression analysis was conducted to determine the anthropometric predictors of systolic blood pressure in the simultaneous context of other covariates. The variables entered into the multiple linear regression models were those that showed an association on bivariate analysis. A p-value of ≤ 0.05 was considered statistically significant. Statistical Package for Social Sciences 16.0 (SPSS) was used for statistical analysis.

## Results

### Sociodemographic characteristics

Three hundred and fifty subjects were contacted in both urban and rural communities of which 335 urban (response rate 95.7%) and 332 rural (response rate 94.8%) subjects responded. There were 163 men and 172 women in the urban population while the rural population consisted of 173 men and 159 women. The mean age ± SD were 43.01 ± 12.8 for the urban dwellers and 43.31 ± 14.91 for rural dwellers. There was no difference in the mean ages of the participant (p = 0.52). There were significantly more rural dwellers that smoked and took alcoholic beverages regularly. The demographic profile as well as alcohol and smoking history of the study population are shown in Table 1.

**Table 1:** Sociodemographic characteristics of the study population

**Variable**	**Rural men n = 173 n (%)**	**Urban men n = 163 n (%)**	**P-value**	**Rural women n = 159 n (%)**	**Urban women n = 172 n (%)**	**P-value**
**Age in years**						
18–20	8 (4.6%)	2 (1.2)		6 (3.8)	2 (1.2)	
21–30	40 (23.1)	24 (14.7)		39 (24.5)	23 (13.4)	
31–40	45 (26.0)	50 (30.7)		53 (33.3)	60 (34.9)	
41–50	21 (12.1)	31 (19.0)		20 (12.6)	48 (27.9)	
51–60	34 (19.7)	37 (22.7)		17 (10.7)	29 (16.9)	
61–70	22 (12.7)	16 (9.8)		12 (7.5)	10 (5.8)	
71–80	3 (1.7)	3 (1.8)		12 (7.5)	3 (0)	
**Marital Status**			0.024			<0.001
Married	143 (82.7)	131 (80.4)		142 (89.3)	120 (69.8)	
Single	28 (16.2)	21 (12.9)		8 (5.0)	22 (12.8)	
Divorced/widowed	2 (1.2)	11 (6.7)		9 (5.7)	30 (17.4)	
**Parity**						<0.001
Nulliparous	--	--		76 (47.8)	34 (19.8)	
1–3	--	--		37 (23.3)	35 (20.4)	
4–7	--	--		44 (27.6)	89 (51.7)	
8–10	--	--		2 (1.3)	14 (8.1)	
**Occupation**			<0.001			<0.001
None	15 (8.7)	16 (9.8)		4 (2.5)	23 (13.4)	
Student	0 (0)	0 (0)		0 (0)	0 (0)	
Applicant	0 (0)	3 (1.8)		0 (0)	1 (0.6)	
Trading	42 (24.3)	66 (40.5)		39 (24.5)	98 (57.0)	
Farmer	93 (53.8)	38 (23.3)		111 (69.8)	15 (8.7)	
Civil servant	23 (13.3)	37 (22.7)		5 (3.1)	32 (18.6)	
Clergy	0 (0)	0 (0)		0 (0)	3 (1.7)	
Chief	0 (0)	3 (1.8)		0 (0)	0 (0)	
**Level of Education**			<0.001			<0.001
None	0 (0)	60 (36.8)		2 (1.3)	76 (44.2)	
Primary	87 (50.3)	34 (20.8)		90 (56.6)	40 (23.3)	
Junior Secondary	40 (23.1)	16 (9.8)		37 (23.3)	8 (4.7)	
Senior Secondary	27 (15.6)	22 (13.5)		26 (16.4)	33 (19.2)	
OND	15 (8.7)	19 (11.9)		4 (2.5)	12 (7.0)	
Bachelor	3 (1.7)	8 (4.9)		0 (0)	2 (1.2)	
Masters	1 (0.6)	4 (2.5)		0 (0)	1 (0.6)	
**Tobacco smoking**			<0.001			<0.001
Smokers	48 (27.7)	14 (8.6)		21 (13.2)	2 (1.2)	
Non-smokers	120 (69.4)	149 (91.4)		136 (85.5)	170 (98.8)	
**Alcohol consumption**			0.207			<0.001
Yes	26 (15)	17 (10.4)		31 (19.5)	2 (1.2)	
No	147 (85.0)	146 (89.6)		128 (80.5)	170 (98.8)	

OND = ordinary national diploma.

### Anthropometric characteristics

Table 2 shows gender specific means for anthropometric measurements in both urban and rural population. Body mass index, waist circumference, waist circumference /height ratio, abdominal height, percentage body fat and BMI were significantly higher in urban men and women than their rural counterparts (p < 0.001). Generally, the mean of all the measured and derived skin fold thicknesses were significantly higher in urban than rural dwellers (p = 0.001) although the abdominal and sub scapular skinfold thickness as well as the SS/TR ratio showed no gender deference among the urban population (p = 0.072, p = 0.36 and p = 0.134).

**Table 2:** Anthropometric and metabolic indices of both population

**Variable**	**Rural men n = 173 (Mean ± SD)**	**Urban men n = 163 (Mean ± SD)**	**P-value**	**Rural women n = 159 (Mean ± SD)**	**Urban women n = 172 (Mean ± SD)**	**P-value**
Weight(*Kg*)	60.87 ± 8.28	67.06 ± 9.43	<0.001	57.93 ± 9.00	70.28 ± 14.05	<0.001
Height(*m*)	1.62 ± 0.07	1.61 ± 0.07	0.41	1.58 ± 0.08	1.57 ± 0.08	3.41
BMI (kg/m^2^)	23.17 ± 2.93	25.70 ± 3.19	<0.001	23.02 ± 3.17	27.83 ± 5.92	<0.001
Waist Circumference (cm)	81.46 ± 7.14	87.69 ± 11.66	<0.001	81.14 ± 7.95	92.36 ± 12.46	<0.001
Abdominal Height (cm)	17.92 ± 2.22	19.36 ± 3.76	<0.001	18.78 ± 2.29	19.85 ± 4.45	<0.001
Waist-height ratio (%)	50.36 ± 5.13	54.01 ± 7.00	<0.001	51.26 ± 5.46	58.07 ± 9.96	<0.001
Skin fold thickness(*mm*)						
Biceps	3.09 ± 1.39	6.89 ± 3.63	<0.001	3.68 ± 2.23	7.98 ± 3.68	<0.001
Triceps	4.89 ± 3.69	10.53 ± 4.51	<0.001	5.62 ± 2.37	12.84 ± 11.05	<0.001
Sub scapular	7.78 ± 1.98	13.01 ± 4.29	<0.001	8.13 ± 2.75	13.44 ± 4.22	<0.001
Abdominal	7.60 ± 3.07	13.44 ± 4.95	<0.001	7.12 ± 2.84	14.38 ± 4.65	<0.001
Superior iliac	7.05 ± 2.94	12.92 ± 4.83	<0.001	6.96 ± 3.11	15.03 ± 5.76	<0.001
∑5sf (mm)^a^	30.43 ± 9.20	48.92 ± 19.70	<0.001	31.52 ± 10.07	59.04 ± 24.04	<0.001
Central SF(mm)	22.44 ± 6.71	33.91 ± 13.43	<0.001	22.21 ± 7.51	39.55 ± 14.34	<0.001
Peripheral SF(mm)	7.99 ± 4.46	15.00 ± 7.55	<0.001	9.30 ± 4.29	19.48 ± 13.61	<0.001
SBP (*mmHg*)	115 ± 14	123 ± 19	<0.001	111 ± 15	122 ± 21	<0.001
DBP (*mmHg*)	74 ± 8	79 ± 13	0.001	71 ± 10	75 ± 14	0.007
RHR	77 ± 11	80 ± 13	0.084	71 ± 11	76 ± 11	<0.001
Fasting glucose (*mmol/l*)	3.56 ± 0.83	3.78 ± 0.25	0.044	3.55 ± 1.14	3.94 ± 2.44	0.067
Cholesterol (*mmol/l*)	3.25 ± 0.86	3.20 ± 0.81	0.604	3.06 ± 084	3.41 ± 1.08	0.001
Triglyceride (*mmol/l*)	1.40 ± 0.37	1.71 ± 0.54	<0.001	1.47 ± 0.43	1.48 ± 0.38	0.94
LDL-C (*mmol/l*)	1.36 ± 0.77	0.94 ± 0.69	<0.001	1.15 ± 0.78	1.24 ± 0.99	0.373
HDL-C (*mmol/l*)	1.24 ± 0.37	1.48 ± 0.46	<0.001	1.23 ± 0.34	1.49 ± 0.43	<0.001

SD = standard deviation, SF = skinfold thickness, ∑5sf = Sum of 5 skinfold thickness, BMI = Body Mass Index.

SBP = systolic blood pressure, DBP = diastolic blood pressure, RHR = resting heart rate, LDL-C = low density lipoprotein cholesterol.

HDL-C = high density lipoprotein cholesterol.

### Blood pressure and resting heart rate characteristics

The mean ± SD of the SBP for the urban and rural population was 122.46 ± 20.80 and 113.33 ± 14.86 respectively while the mean ± SD of the DPB was 76.58 ± 15.03 and 73.22 ± 9.78 for urban and rural population respectively (Table 2). Both SBP and DBP differed significantly between the two populations (p < 0.001) although DBP was not significantly different between urban and rural men. Hypertension was significantly prevalent among the urban than the rural population. (Table 3) The mean ± SD of the resting heart rate was 78.10 ± 12.37/minute for the urban and 74.80 ± 11.56/minute for the rural dwellers. This difference was also statistically significant (p < 0.001). See Table 2.

**Table 3:** Prevalence of cardiovascular risk factors

**Variable**	**Rural (n = 332) (n/%)**	**Urban (n = 335) (n/%)**	Statistics*			
			**χ**^**2**^	**RR**	**95% CI**	***P*-value**
Hypertension	16 (4.8)	58 (17.3)	26.39	0.87	0.82-0.92	<0.001
Diabetes	3 (0.9)	9 (2.7)	3.0	0.33	0.09-1.23	0.083 (NS)
IFG	2 (0.6)	5 (1.5)	71.30	0.40	0.077-2.07	0.451 (NS)^§^
Overweight	60 (18.1)	164 (49.0)	46.64	0.62	0.55-0.70	<0.001
Obesity	9 (2.7)	61 (18.2)	2.78	0.84	0.79-0.88	<0.001
Dyslipidaemia	156 (47.0)	136 (40.6)	3.13	0.86	0.73-1.03	0.096 (NS)
Cholesterol	15 (4.5)	7 (2.1)	9.14	2.23	0.898-5.54	0.077 (NS)
Triglyceride	72 (21.8)	108 (32.1)	0.97	0.59	0.415-0.83	0.003
LDL-C	11 (3.3)	7 (2.1)	0.041	1.62	0.619-4.22	0.323 (NS)
HDL-C	88 (26.6)	87 (25.9)	21.83	1.04	0.734-1.46	0.839 (NS)
Alcohol	57 (17.2)	19 (5.7)	40.27	0.33	0.20-0.54	<0.001
Smoking	69 (20.8)	15 (4.5)	43.23	0.21	0.13-0.37	<0.001

* comparing proportion of urban and rural dwellers in each group.

RR-Relative risk, CI- confidence interval; IFG-Impaired fasting glucose, §-fisher’s exact p-value.

NS-Not significant.

### Metabolic characteristics

The mean serum TC and HDL-C were significantly higher in urban women compared to rural women (p = 0.001 and p = <0.001) while there were no significant differences in the mean triglycerides and LDL-C between the rural and urban women. Significantly higher level of TG and HDL-C (p = <0.001, p = <0.001) were also noted among the urban compared to rural men although the mean LDL-C was significantly higher among rural men (p = <0.001). The mean serum glucose level was significantly higher in both genders of the urban compared to the rural population (p = 0.01).

### Prevalence of cardiovascular risk factors

Table 3 shows the prevalence of cardiovascular risk factors in the two populations. Hypertension, overweight and obesity were significantly prevalent among the urban population (p < 0.001) while there was no significant difference in the prevalence of diabetes and dyslipidaemia between the two populations although hypertriglyceridaemia was significantly prevalent among the urban population (p = 0.003). Alcohol use and smoking however, were more prevalent among the rural population (<0.001).

### Correlation

Tables 4 and 5 show the correlations of the anthropometric parameters with the cardio-metabolic parameters in women and men respectively. Age had a significant positive correlation with SBP (r = 0.222, p = <0.001) and DBP(r = 0.179, p = <0.001) in the total population although; in the sub-population of urban and rural men, the correlation with SBP was not significant (p = 0.117 and p = 0.190). Systolic blood pressure was positively correlated with all anthropometric variables (Table 4) except BMI in rural women (0.054), and abdominal height in rural men (p = 0.351). The diastolic blood pressure had a significant correlation with most anthropometric parameters except %BF and abdominal height in rural men. Among the women cohort, diastolic blood pressure had a significant positive correlation with most anthropometric parameters except %BF in urban women and abdominal height in rural women (Table 5). There was an inverse correlation of HDL-C with BMI, WC, WCHR and RHR in rural men but only significantly so with BMI and WCHR (p = 0.013 and 0.011).

**Table 4:** Spearman correlation of anthropometric indices with mean blood pressure, lipids and fasting blood glucose level among the men cohort

		**Age**		**BMI**		**WC**		**WCHR**		**ABDht**		**%BF**	
		**Urban**	**Rural**	**Urban**	**Rural**	**Urban**	**Rural**	**Urban**	**Rural**	**Urban**	**Rural**	**Urban**	**Rural**
Systolic BP	*r*	.123	.100	.236*	.233*	.387*	.355*	.344*	.370*	.474*	-.071	.078	.250*
	*p*	.117	.190	.002	.002	<.001	<.001	<.001	<.001	<.001	.351	.320	.001
Diastolic BP	*r*	.203*	.092	.155*	.054	.353*	.155*	.320*	.154*	.412*	.019	-.007	.066
	*p*	.009	.230	.048	.479	<.001	.042	<.001	.044	<.001	.808	.931	.391
RHR	*r*	-.094	-.037	.135	.182*	.085	.264*	.085	.265*	.288*	-.030	-.065	.116
	*p*	.234	.632	.087	.017	.281	<.001	.281	<.001	<.001	.694	412	.128
Glucose	*r*	-.055	-.020	.056	.138	.027	.132	.054	.146	.055	-.039	-.028	.096
	*p*	.285	797	.476	.070	.733	.083	.497	.055	.489	.611	.722	.207
Cholesterol^§^	*r*	.178	-.061	.055	-.037	-.006	-.037	.082	-.035	.029	.115	.054	-.058
	*p*	.023	.429	.483	.633	.944	.628	.298	.648	.717	.131	.490	.451
Triglyceride	*r*	-.047	.059	.029	.059	.056	.075	.012	.031	-.072	-.012	-.016	.088
	*p*	.553	.441	.712	.438	.481	.327	.879	.683	.358	.875	.842	.250
HDL-C	*r*	.148	.009	.070	-.189	-.040	-.145	.048	.194*	-.036	.051	-.048	-.143
	*p*	.059	.904	.375	.013	.612	.058	.547	.011	.649	.503	.547	.061
LDL-C	*r*	.175*	-.104	-.026	.028	.000	.001	.047	.032	.070	.121	.125	-.034
	*p*	.026	.175	.738	.717	.998	.994	.550	.672	.375	.112	.111	.660

SBP (systolic blood pressure), DBP (diastolic blood pressure), RHR (resting heart rate), LDL-C (low density lipoprotein cholesterol).

HDL-C (high density lipoprotein cholesterol), § (Total cholesterol), BMI = body mass index, WC = waist circumference.

WCHR = waist circumference height ratio, ABDht = abdominal height, %BF = percentage body fat.

r = spearman correlation coefficient, p = p-value, * = significant.

**Table 5:** Spearman correlation of anthropometric indices with mean blood pressures, lipids and fasting blood glucose level among the women cohort

		**Age**		**BMI**		**WC**		**WCHR**		**ABDht**		**%BF**	
		**Urban**	**Rural**	**Urban**	**Rural**	**Urban**	**Rural**	**Urban**	**Rural**	**Urban**	**Rural**	**Urban**	**Rural**
Systolic BP	*r*	.285*	.293*	.270*	.153	.276*	.369*	.253*	.388*	.290*	-.115	.050	.380*
	*p*	<.001	<.001	<.001	.054	<.001	<.001	.001	<.001	<.001	.150	.512	<.001
Diastolic BP	*r*	.129	.237*	.258*	.201*	.264*	.315*	.236*	.323*	.158*	-.040	.066	.358*
	*p*	.092	<.001	.001	.011	<.001	<.001	.002	<.001	.038	.619	.392	<.001
RHR	*r*	-.004	.028	.189*	.288*	.153	.465*	.262*	.302*	.084	.176*	.141	.194*
	*p*	.956	.725	.013	<.001	.045	<.001	.001	<.001	.272	.026	.066	.014
Glucose	*r*	.118	.126	.195*	.050	-.080	.258*	-.059	.204*	-.133	-.042	.090	.148
	*p*	.123	.113	.010	.534	.298	.001	.444	.010	.083	.602	.241	.062
Cholesterol^§^	*r*	-.235*	.010	-.122	.106	-.092	.081	-.142	.090	-.063	.001	.090	.091
	*p*	.002	.905	.111	.184	.231	.313	.064	.260	.412	.991	.239	.253
Triglyceride	*r*	.004	.056	-.031	.082	-.028	.169*	-.082	.137	.050	-.113	.041	.127
	*p*	.954	.481	.686	.303	.712	.034	.284	.085	.513	.155	.591	.110
HDL-C	*r*	-.124	-.131	-.035	-.044	-.125	-.102	-.072	-.059	-.035	.028	.021	-.105
	*p*	.106	.10	.648	.583	.103	.203	.350	.457	.648	.729	.781	.189
LDL-C	*r*	-.190	.067	-.111	.097	-.030	.080	-.101	.089	-.057	.017	.075	.121
	*p*	.013	.401	.148	.225	.698	.318	.186	.264	.461	.830	.329	.130

SBP (systolic blood pressure), DBP (diastolic blood pressure), RHR (resting heart rate), LDL-C (low density lipoprotein cholesterol).

HDL-C (high density lipoprotein cholesterol), § (Total cholesterol), BMI = body mass index, WC = waist circumference.

WCHR = waist circumference height ratio, ABDht = abdominal height, %BF = percentage body fat.

r = spearman correlation coefficient, p = p-value, * = significant.

### Regression

In a multivariate regression analysis, the impact of Age, BMI, %BF, abdominal height, WCHR and WC were evaluated with systolic blood pressure (Table 6). Age appeared to be the most significant predictor of SBP in rural women (β = 0.258, R^2^ = 0.157, p =0.016) and urban women (β = 0.351, R^2^ = 0.157 p <0.001). Although WC appeared to be a predictor of SBP in rural men (β =0.273, R^2^ = 0.064, p = 0.003), this effect became insignificant when Age, %BF WCHR were added to the model. Among urban men, abdominal height was the only significant predictor of SBP (β =0.281, R^2^ = 0.16, p = 0.001) Table 6 shows the table of regression.

**Table 6:** Summary of multiple regression analysis for anthropometric variables predicting systolic blood pressure in the study population

**Variable**	**Explanatory variables**	**Adjusted R**^**2**^	**β coefficient**	**Standard error**	**P-value**
(*Rural Women)*					
Model1: Age, WC, WCHR, %BF.	Age	0.157	0.258	0.104	0.016
(*Urban Women*)					
Model1: WC, BMI, Age, WCHR, Abdht.	Age	0.201	0.351	0.140	<0.001
	BMI	--	0.593	0.640	0.001
	WCHR	--	-.480	0.402	0.008
(*Urban Men*)					
Model1: Age, WC, BMI, WCHR, AbdHt	AbdHt	0.16	0.281	0.423	0.001
(*Rural Men*)					
Model1: BMI, WC,	WC	0.064	0.273	0.178	0.003
Model2: BMI, WC, Age, WCHR, %BF	--	0.068	--	--	--

WC; waist circumference, BMI; body mass index, %BF; percentage body fat; WCHR; waist circumference height ratio, Abdht; abdominal height.

## Discussion

This epidemiological study provides distribution and correlation of anthropometric and metabolic cardiovascular risk factors in urban and rural residents of two Nigerian communities with similar extraction but different environmental/socioeconomic modifier. The result showed significantly higher level of body fat percentages, BMI, WC, WCHR, CSF and PSF among the urban population compared to their rural counterparts. There is in effect a significantly higher prevalence of overweight, obesity and hypertension in the urban samples. It is likely that these variations in the cardio-metabolic profile of the two populations are due to the disparity in their lifestyles and dietary preferences. The rural participants were majorly subsistence farmers and manual unskilled workers while urban dwellers in this study were mainly traders and civil servants who were less physically active than their rural counterparts.

This finding is in accord with our earlier studies and that of other authors reflecting the effect of westernization on anthropometric profiles as well as CVD risks in urban populations [15,27,28]. The nutritional, socioeconomic as well as epidemiological transition in different developing countries has been the explanation for this differing profile between rural and urban population [27,28]. Nutritionally, the urban dietary lifestyles favours the shift from traditional, naturally occurring diets with little or no variation to packaged and processed foods rich in animal-source, fat, salt and sugar since the latter are readily available and affordable. Another influence of westernization and improved social and economic factors is the reduction of physical activities amongst the urban dwellers. This reduction in physical activities is fostered by the presence of available and affordable transportation systems, sedentary jobs, remote-controlled and automatic appliances. This lifestyle is contrasted by the pattern among rural residents in which long distances are walked; vigorous activities are carried out in farming and other occupations which are largely manual. In addition, rural residents eat less of processed food since their foods are locally obtained. We observed that there was no significant difference in the prevalence of dyslipidaemia between the two populations. The prevalence of dyslipidaemia observed in this study was largely driven by high triglycerides and low HDL in both populations; although hypertriglyceridaemia was significantly prevalent among the urban population. The higher prevalence of overweight and obesity among the urban populations could have accounted for the significantly higher prevalence of hypertriglyceridaemia.

Women generally have more body fat than men hence, the observed higher mean value for most anthropometric indices. In the same vein, the women in this study showed higher mean value of SBP and DBP compared to the men. However, we observed that there was no gender difference in the mean value of the WC and CSF among the rural population suggesting the same degree of abdominal adiposity in both gender of this population. It is likely that the presence of more female farmers and lesser female civil servant in this population accounted for this observation although it could have been expected that other adiposity indicators should show this same trend.

We also observed as in the studies by Mistra et al. [10], the significant linear associations of SBP and DBP with age and the anthropometric parameters as well as adiposity indicators considered for this study: WC, BMI, BF%, WCHR, abdominal height and the skin fold thicknesses (Tables 4 and 5, p < 0.001 to p < 0.047). However, in rural women, abdominal height did not correlate with SBP and BMI did not correlate with DBP. This trend may be due to the fact that rural women were generally less obese than their urban counterparts. Although age was an independent predictor of SBP in the in the female population, its effect was not significant in the sub-population of urban and rural men. This could reflect the fact that though blood pressure increases with age, the effect of age may be masked in the presence of other atherosclerotic risk factors; reflecting a higher cumulative exposure to other risks as age increases. This observation was also made by Dolls et al. [29]. Following multiple regression, age was found to be the most important predictor of high systolic blood pressure among women although BMI and WCHR were equally important among urban women.

This study is however limited by the fact that we did not define the socioeconomic status as well as the dietary habits of both the urban and rural dwellers hence we could only hypothesize that the anthropometric differences noted in these two populations were likely due to dietary as well as lifestyle differences. A further study to evaluate the dietary pattern, socioeconomic status and the degree of exercise of these two populations will address these concerns.

## Conclusion

In conclusion, this study showed that many cardiovascular risk factors such as overweight, obesity, hypertension and higher anthropometric indices were significantly prevalent among the urban population compared to the rural population while smoking and alcohol intake were more prevalent among the rural population. These outlooks suggest a linkage between dietary and environmental influence on the anthropometry and cardiovascular status of these two Nigerian communities of the same ancestry. Appropriate health education and lifestyle modification strategies may reduce the increased burden of cardiovascular risk factors associated with rural–urban migration.

## Competing interests

None of the authors declare any financial or non-financial potential conflicts of interest, including funding from or shares from organizations that stand to gain or lose from the publication of this manuscript, or any other competing interests that may cause embarrassment were they to become public after the publication of the manuscript.

## Authors’ contributions

OSA conceived the idea of, the design and execution of the study. PBA analyzed and interpreted the data and drafting the manuscript. AAA contributed to the interpretation of the data. OSA, PBA and AAA contributed to the literature search and to the writing of the manuscript. All authors read and approved the final manuscript.
